# Biometric indices of *Scylla serrata* (Forskal, 1775): Exploring gender-specific growth patterns from the Cochin Estuary, southwest coast of India

**DOI:** 10.1016/j.heliyon.2025.e42829

**Published:** 2025-02-19

**Authors:** K.A. Aneesa, E.R. Chaithanya, Sijo P. Varghese

**Affiliations:** a Department of Marine Biology, Microbiology and Biochemistry, School of Marine Sciences, Fine Arts Avenue, Cochin University of Science and Technology, Cochin, 682016, Kerala, India; b Fishery Survey of India, Cochin Zonal Base, Kochangady, Cochin, 682005, Kerala, India

**Keywords:** Allometric growth, Carapace width-weight relationship, Condition factors, Kerala, Mud crab, Size frequency distribution, *Scylla serrata*

## Abstract

The present study investigated the biometric indices and gender-specific growth patterns of the mud crab *Scylla serrata,* a commercially important crab species from the Cochin Estuary, southwest coast of India. Biometric indices include size-frequency distribution, carapace width-weight relationship (CW-WR), and condition factors (Fulton's condition factor (K), Allometric condition factor (K*a*), and relative condition factor (K*n*)), which are crucial in assessing the biological changes in crabs. The CW-WR and interrelationships between various morphometric characters were estimated using 4022 crabs comprising 2048 males and 1974 females of *S. serrata* systematically collected from June 2020 to May 2023. Statistical analyses were employed to derive CW-WR for male and female crabs and pooled data. The results indicated a significant correlation between carapace width and weight, revealing potential sex-specific variations in the growth patterns of male *S. serrata*, expressed as W = 0.0000447 × CW^3.268^. At the same time, for females, it is W = 0.0000281∗CW^2.86^. Additionally, the relationship for the pooled data, combining both males and females, was W = 0.000103 × CW^3.084^. The '*b*' values for CW-WR in males and females were 3.268 and 2.86, respectively, the '*b*' values of CW-WR revealed potential gender-specific variations in the growth patterns i.e. Positive allometric growth in males and negative allometric growth in females of *S. serrata*. The allometric relationships between characters were positive and highly significant. Fulton's condition factor (K) ranged from 0.001 to 0.052 in females and 0.002 to 0.054 in males. The allometric condition factor (K*a*) ranged from 0.0031 to 0.090 in females and from 0.007 to 0.016 in males. The relative condition factor (K*n*) ranged from 0.113 to 3.205 in females and from 0.152 to 3.516 in males. This study provides information on the growth and condition of crabs, which will help identify effective fisheries management strategies and contribute to a broader understanding of *S. serrata* biology.

## 1 Introduction

The Cochin Estuary is the largest brackish water ecosystem in Kerala and is part of the Ramsar Site along the southwest coast of India. This is a potential source for mud crab production. Mud crabs are commercially essential portunid crabs primarily found in mangroves across the tropical and temperate zones of the Pacific and Indian Ocean regions [1]. These crabs, often called mangrove crabs, are associated with intertidal and subtidal areas within the mangrove ecosystem. There are four species of mud crab in the genus Scylla: *Scylla serrata*, *Scylla olivacea*, *Scylla paramamosain*, and *Scylla tranquebarica* [[1], [2], [3], [4]]. Kerala is an important state that contributes to India's inland crab fishery and depends on *S. serrata* and *S. olivacea* [5]. India is the world's largest exporter of crabs; the top destinations are China, the US, and Singapore. India's crab exports during 2021-22 were 6938 MT, showing an increase of 25.94 % compared to 2020–21 [6]. This increasing demand for mud crabs may lead to increased exploitation [1] and threaten the well-being of the population. Estimation and analysis of biometric indices are crucial for the management of these resources because they can contribute valuable information on crab population growth and well-being [7]. Commonly studied biometric indices of crabs include size-frequency distribution, length-weight (carapace width-weight) relationship, condition factors, and sex ratio [8]. Biometric analysis provides strong support for stock identification approaches and is vital for estimating the population size of stocks for exploitation purposes. The size-frequency distribution of a fish or crab population can determine the distribution of individuals in terms of size class and is a popular method for evaluating life history [9]. Carapace width-weight relationships (CW-WR) are useful when measuring body length, but are more difficult or time-consuming than measuring the weight of aquatic species [10]. In such instances, length can be estimated from the measured weight data. Length-weight relationships have been employed in various fisheries ecological studies and condition estimations [11]. CW-WR vary among mud crab species based on inherent body shapes and physiological factors, including maturity and spawning [12]. This relationship can fluctuate throughout the season or within a few days [13]. Therefore, it is an essential tool in fishery science and is often used as a proxy to gauge the health of a population, assuming that a higher mass at a given length reflects better health [[14], [15], [16], [17]]. Condition factors indicate ﬁsh well-being, population health [18], environmental quality, and suitability [19]. These indices indicate the interaction of abiotic and biotic components in ﬁsh physiology [20]. There are three widely used condition factors: Fulton's condition factor (K) [21], allometric condition factor (K*a*) [22], and relative weight condition factor K*n* [19]. K values indicate individuals in the form of hypothetical fish. Ka values indicate deviations from the form of hypothetical fish. K*n* calculates variations from an average weight of length [18], which is useful for the sustainable management of fishery resources [9]. CW-WR and the condition of crabs are influenced by a range of factors, including food availability and water quality, as well as the size, age, and sexual maturity of a crab. According to the available literature, several researchers have explored the length-weight relationship of *S. serrata*. Among notable studies are [[23], [24], [25], [26]]. The fishery and biology of the crabs of the Kakinada region were studied [27], and the exploitation and fishery of *S. serrata* from the Korapuzha estuary in Kerala were reported [28]. However, only [29,30] examined the Fulton and relative condition factors with the CW -WR of *S. serrata.* These have been largely confined to specific regions like the Kakinada and Korapuzha estuaries. In contrast, the Cochin Estuary, which serves as a critical habitat and fishery resource for *S. serrata*, remains understudied. Although several biometric studies are available for mangrove ﬁsh species and crustaceans [7,8,31,32], very few have investigated *S. serrata* in Indian waters. The population of *S. serrata* from the Cochin estuary remains understudied regarding the biometric indices, particularly in integrating LFDs, CW-WR, and condition factors (K, K*a*, K*n*). The lack of such studies limits understanding of growth patterns, population structure, and health in the estuary, which is essential for sustainable management. Therefore, we attempted to estimate and discuss the length-frequency distributions (LFDs), Carapace width-weight relationshipsCW-WR), and condition factors (K, K*a*, and K*n*) of *S. serrata* inhabiting the Cochin estuary.

## 2 Materials and methods

### 2.1 Study area and sample collection

**Fig. 1:**
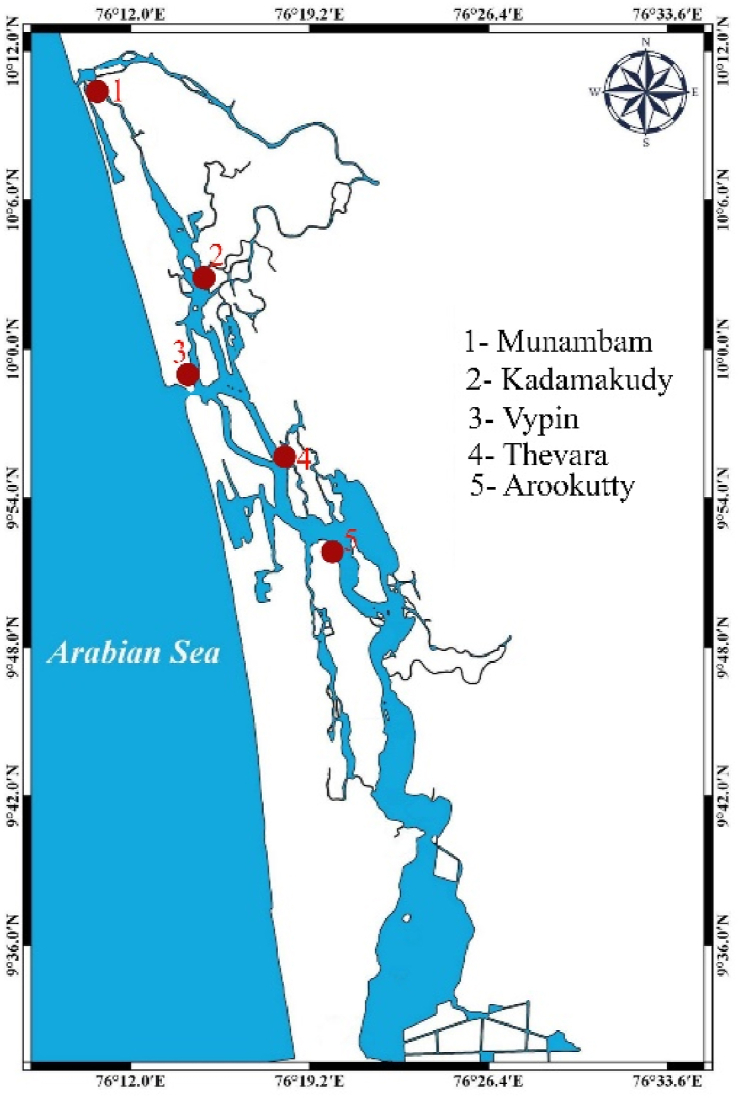
Sampling site.

*S. serrata* samples were collected from the Cochin estuary, which is a tropical positive estuarine system extending between 9°40 and 10°12 N and 76°10 and 76° 30′E [5]. The present study was conducted over 3 years (from June 2020 to May 2023) from active crab landing areas named Munambam, Kadamakkudy, Vypin, Thevara, and Arookutty (Fig. 1). Sampling was carried out monthly during peak fishing activity at each site to ensure representative data across temporal and spatial scales. A total of 4022 specimens comprising 2048 male and 1974 female crabs were collected during the study period. Crabs were obtained from local mud crab middle traders mainly caught by crab gillnets and ring nets [5]. Crabs were identified at the species level based on taxonomic keys [1] and sexed into males and females. Measurements were made for carapace width (CW), carapace length (CL), and total weight(W), which were measured separately for males and females. In addition, the width and length of the abdomen (AW and AL) were recorded for females and the chelar (propodus) length and width (ChL and ChW) were recorded for males. Crabs were classified according to CW: CW > 100 mm, juveniles; 100–149 mm, sub-adults; and CW < 149 mm, adults [33]. A Vernier caliper (accuracy, 0.02 mm) was used for the length measurements, and the total weight of the crab was determined to the nearest gram using a digital balance.

### 2.2 Population structure

All data were recorded in a spreadsheet and analysed using R software (R-4.3.2). The length frequency distribution was studied to investigate the age, growth, and population structure of the target species. The population of *S. serrata* length frequency distributions (LFDs) was determined by binning the carapace width (CW) into 10 mm class intervals and plotted to visualize population structure using the R software.

### 2.3 Carapace width-weight relationships (CW-WR)

(1) W = *a*L^*b*^ [19]

was used to calculate the CW-WR. Using the linear form, that is, the log-transformed equation is

(2) log(W)=log(a)+blog(L)

W is the weight of the crab and L is the carapace width of the crab (CW). The parameters ‘*a*’ represent the intercept and ‘*b*’ the slope of the relationship. Where *a* and *b* were estimated through nonlinear regression analysis in R. The regression models were fitted in R, and the summary(.) function was used to extract the coefficients, their standard errors, t-values, and associated p-values. These outputs were used to evaluate the significance of the relationships and determine the strength of the fit.

The growth of crabs was assessed as isometric if *b* = 3 [21]. When *b* < 3, the crab's growth is estimated as negatively allometric, whereas when *b* > 3, the crabs become heavier and are estimated to show positive allometric growth.

### 2.4 Interrelationships between different morphometric characters

(3) Linear equation Y = a + b X

was used to calculate the interrelationships between different morphometric characters.

Y = Dependent Variable, X = Independent variable, a = intercept and b = slope.

CW and CL were taken as independent variables, and W, ChL, and ChW in males and W, AW, and AL in females were taken as dependent variables. Correlation coefficient (r) values were calculated.

### 2.5 Condition factors

Fulton's condition factor (K), allometric condition factor (K*a*), and relative condition factor (K*n*) were used to examine the growth and physical condition of an organism. Condition factors were calculated to assess the health and well-being of the crabs. Statistical differences between sexes and size classes were assessed using regression coefficients and their significance.

#### 2.5.1 Fulton's condition factor (K)

The coefﬁcient of the condition factor, or Fulton's condition factor(K), was calculated as

(4) K = 100 × W/L^3^ [21]

Where W is the total weight, and L is the carapace width (CW). When using weight in grams and length in millimetres for calculating K, the constant value was 100. This constant is a scaling factor that ensures that the condition factor is dimensionless and standardised for comparison. K is used to study the well-being of an organism, and if the growth is allometric, K is often used [18].

#### 2.5.2 Allometric condition factor (K*a*)

(5) K*a* = 100 × W/L^*b*^ [22]

where W is the weight and Lis the organism's length; in the case of the crab, CW is taken as the length, and *b* is the value from the CW-WR.

#### 2.5.3 Relative condition factor (K*n*)

(6) K*n* = W_o_/W_c_ [19]

was used to assess the mud crab conditions. Where W_o_ is the observed weight and W_c_ is the calculated weight. When K*n* ≥ 1, it indicates a good growth condition of the crab, and when K*n* < 1, it is deduced that the organism is in poor growth conditions.

## 3 Results

### 3.1 Population structure

**Fig. 2:**
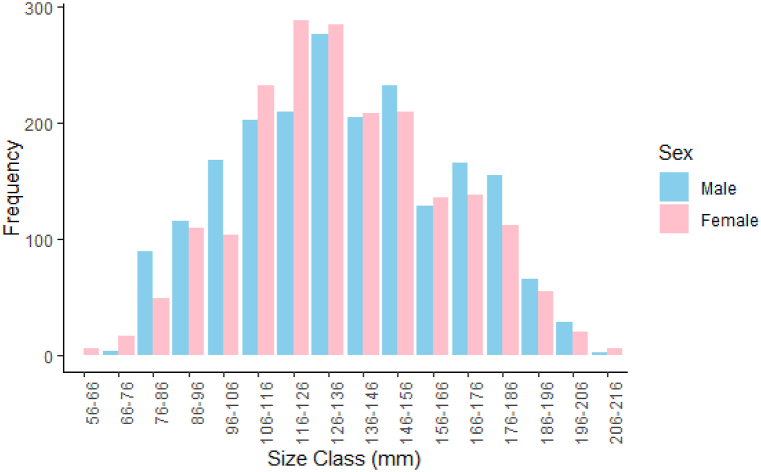
Size frequency distribution of *S. serrata* male and female.

A total of 4022 *S. serrata* specimens, comprising 2048 male and 1974 female crabs, were collected during the study. The size-frequency distributions of males and females revealed that the dominant size groups in the fishery were male and female crabs 126–136 mm and 116–126 mm size classes, respectively (Fig. 2, Table 1). The minimum and maximum CW ranged from 56 mm to 214 mm and 50–1700 g, respectively, for females, whereas for males, CW ranged from 73 mm to 209 mm and weight ranged from 50 g to 1955 g.

**Table 1:** Mean and Standard Deviation of CW, CL, and W regarding *S. serrata* measurements.

sex	number	Carapace width (CW) range(mm)	Mean CW	Carapace length (CL) range	Mean CL	Weight range	Mean weight
Female	1974	56–214	135.04 ± 28.75	32–145	89.41 ± 18.75	50–1700	409.33 ± 250.85
Male	2048	73–209	136.59 ± 30.29	27–195	91.63 ± 22.63	50–1955	513.53 ± 365.99
Pooled	4022	56–214	135.83 ± 29.55	27–195	90.54 ± 20.84	50–1955	462.39 ± 319.03

### 3.2 Carapace width-weight relationships (CW-WR)

**Fig. 3:**
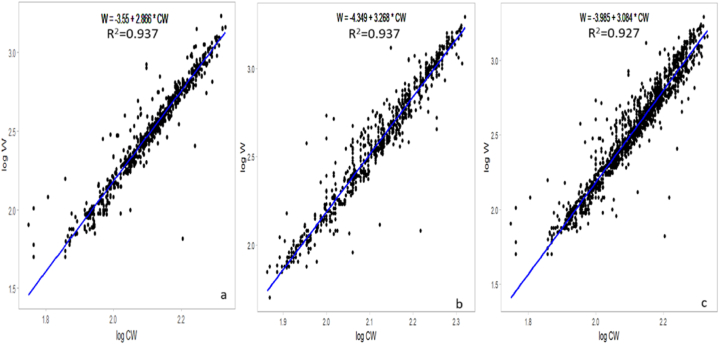
Carapace width-weight relation of *S*. *serrata* in female (a) male (b) pooled (c).

**Fig. 4:**
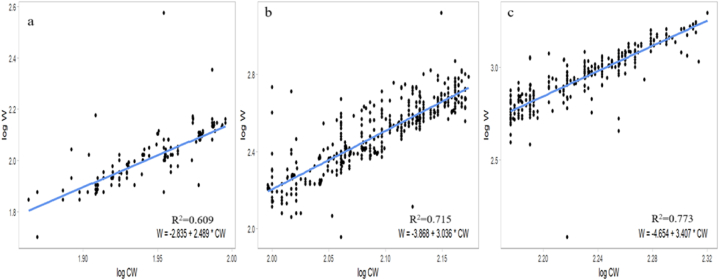
Carapace width-weight relation of *S. serrata* male juvenile (a), sub-adult (b), and adult (c).

**Fig. 5:**
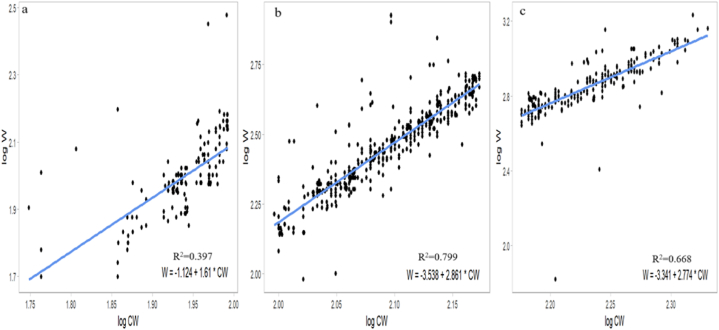
Carapace width-weight relation of *S. serrata* female juvenile (a), sub-adult (b), and adult (c).

**Fig. 6:**
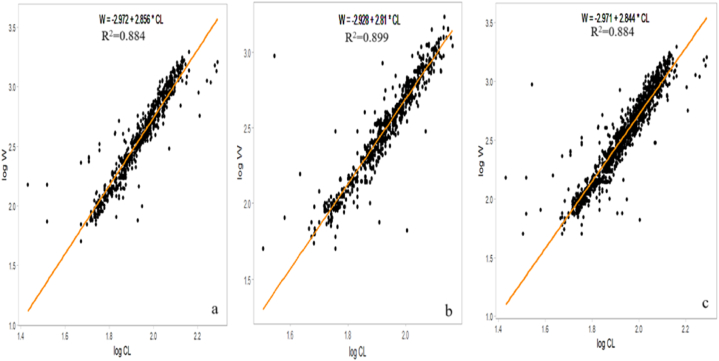
Carapace length-weight relation of *S. serrata* in male (a), female (b), pooled (c).

The results indicated a significant correlation between CW and W, revealing potential sex-specific variations in the growth patterns of *S. serrata*; for males, the CW-WR established was W = 0.0000447∗CW^3.268^, while that for females was W = 0.000281∗CW^2.86^. Additionally, the relationship for the pooled data, combining both males and females, was W = 0.000103 ∗CW^3.084^. The *b* values for males and females were *b* = 3.268 and *b* = 2.86, respectively, where males were positive and females showed negative growth allometry. The *b* value differed significantly (P = 0.000002) from that of isomerism (*b* = 3). The estimated *b* also indicated a higher increment rate of weight in males than in females of *S. serrata*. A scatter diagram for females, males and pooled was constructed by plotting W against the CW of individual crabs (Fig. 3) to discern these sex-related variations in the CW-WR. A strong positive correlation (R^2^ > 0.93) was observed between CW and W for *S. serrata* (Table 2). In male juvenile crabs, the CW-WR was established as W = 0.001462∗CW^2.489^, for the subadults W = 0.000136∗CW^3.036^^,^ and the adults W = 0.0000222∗CW^3.407^ (Table 3). The scatter diagram for juvenile, sub-adult, and adult male crabs was constructed by plotting W against the CW of the crabs (Fig. 4) to spot variations in the CW-WR. In juvenile male crabs, *b* = 2.489, indicating negative allometric growth. However, in sub-adults and adults, *b* = 3.036 and *b* = 3.407, respectively, indicated positive allometric growth. In female juvenile crabs, the CW-WR established was W = 0.07516229∗CW^1.61^ for the subadults W = 0.00028973∗CW^2.861^ and the adults W = 0.00045604∗CW^2.774^ (Table 4). The scatter diagram for juvenile, sub-adult, and adult female crabs was constructed by plotting W against the CW of the crabs (Fig. 5) to distinguish the variations in the CW-WR. In juvenile female crabs, *b* = 1.61, showing negative allometric growth; in sub-adults and adults, *b* = 2.861 and *b* = 2.774, respectively, indicating negative allometric growth. The carapace length-weight relationships (CL-WR) for males W = −2.972 + 2.856∗CL for females is W = −2.928 + 2.81 ∗CL(Fig. 6) the ‘*b*’ was 2.856 and 2.81, respectively. The results showed a significant deviation from the isometric growth patterns. The high R^2^ values indicate that a large proportion of the variance in the dependent variable is explained by the independent variable. The CL-WR for pooled data W = −2.971 + 2.844∗CL (Fig. 6) in the pooled data results showed a substantial deviation from the isometric growth pattern.

**Table 2:** Regression equations and R^2^ values of *S. serrata*.

sex	n	Regression Equation	W = aCW^b^	a	b	R^2^	allometry
Female	1974	W = −3.55 + 2.866∗CW	W = 0.000281∗CW^2.866^	0.000281	2.866	0.937	-ve
Male	2048	W = −4.349 + 3.268∗CW	W = 0.0000447∗CW^3.26^	0.0000447	3.268	0.937	+ve
Pooled	4022	W = −3.985 + 3.084∗CW	W = 0.000103∗CW^3.084^	0.000103	3.084	0.927	+ve

**Table 3:** Regression equations and R^2^ values of male *S. serrata*.

Male	Regression Equation	W = aCW^b^	a	b	R^2^	Allometry
Juvenile	W = −2.835 + 2.489∗CW	W = 0.001462∗CW^2.489^	0.001462	2.489	0.609	-ve
Subadult	W = −3.868 + 3.036∗CW	W = 0.000136∗CW^3.036^	0.000136	3.036	0.715	+ve
Adult	W = −4.654 + 3.407∗CW	W = 0.0000222∗CW^3.407^	0.000022	3.407	0.773	+ve

**Table 4:** Regression equations and R^2^ values of female *S. serrata*.

Female	Regression Equation	W = aCW^b^	a	b	R^2^	Allometry
Juvenile	W = −1.124 + 1.61∗CW	W = 0.07516229∗CW^1.61^	0.075162	1.161	0.397	-ve
Subadult	W = −3.538 + 2.861∗CW	W = 0.00028973∗CW^2.861^	0.000289	2.861	0.799	-ve
Adult	W = −3.341 + 2.774∗CW	W = 0.00045604∗CW^2.774^	0.000456	2.774	0.668	-ve

### 3.3 Interrelationships between different morphometric characters

**Fig. 7:**
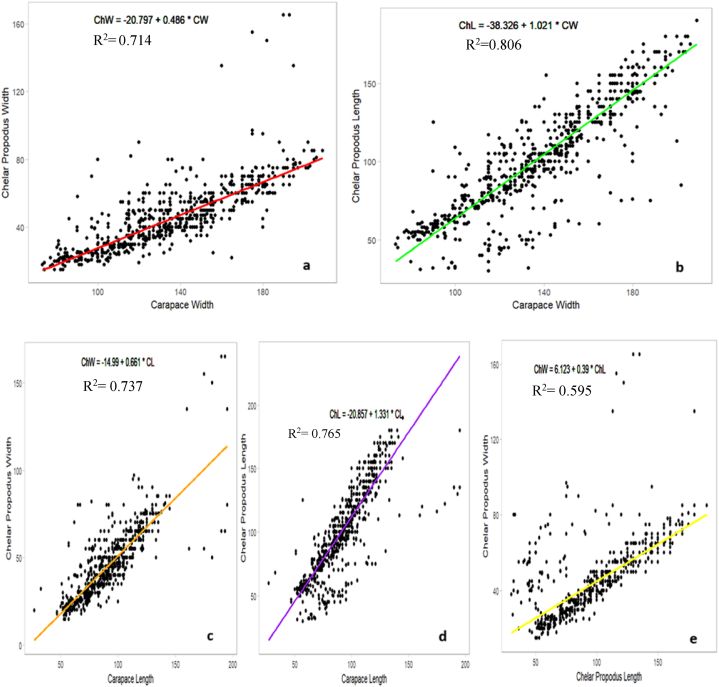
Carapace width-Chelar propodus width relation (a), Carapace width-Chelar propodus length relation (b), Carapace length-Chelar propodus width relation (c), Carapace length-Chelar propodus length relation (d), Chelar propodus width -Chelar propodus length relation (e) of male *S. serrata*.

The interrelationships between different morphometric characteristics of male and female *S. serrata* are presented in Table 5, Table 6, respectively. Interrelationships between the set of characters of males (Fig. 7) studied, CW strongly correlates with ChL and ChW, accounting for 80.63 % and 71.42 % of their variations, respectively. CL also showed good predictive capability for chelar propodus dimensions, explaining 76.54 % and 73.77 % of the variations in ChL and ChW, respectively. ChL and ChW are also moderately correlated with 59.46 % of the variation in ChW explained by ChL. Carapace dimensions are reliable indicators for W and chelar propodus dimensions. The present study suggested that the relationship was mostly positive, and strong R^2^ values and low p-values indicated that these correlations were statistically significant.

**Table 5:** Allometric equations and correlation coefficients (r) values between different variables in male *S. serrata*.

Independent variable (x)	Dependent variable (y)	Allometric growth equation (y = a + bx)	R^2^ value	P value
Carapace length	Weight	W = −2.972 + 2.856∗CL	0.884	0
Carapace width	Chelar propodus length	ChL = −38.326 + 1.021 ∗CW	0.806	0
Carapace width	Chelar propodus width	ChW = −20.796 + 0.485 ∗CW	0.714	0
Carapace length	Chelar propodus length	ChL = −20.857 + 1.331 ∗CL	0.765	0
Carapace length	Chelar propodus width	ChW = −14.990 + 0.660∗CL	0.737	0
Chelar propodus length	Chelar propodus width	ChW = 6.1228 + 0.3896 ∗ ChL	0.595	0

**Table 6:** Allometric equations and correlation coefficients (r) values between different variables in female *S. serrata*.

Independent variable (x)	Dependent variable (y)	Allometric growth equation (y = a + bx)	R^2^ value	P value
Carapace length	Weight	W = −2.928 + 2.81 ∗CL	0.899	0
Carapace width	Abdominal length	AL = −11.744 + 0.493 ∗CW	0.658	0
Carapace width	Abdominal width	AW = −18.746 + 0.63 ∗CW	0.828	0
Carapace length	Abdominal length	AL = −13.151 + 0.76 ∗CL	0.665	0
Carapace length	Abdominal width	AW = −18.995 + 0.953∗CL	0.809	0
Abdominal length	Abdominal width	AL = 12.489 + 0.638 ∗ AW	0.528	0

**Fig. 8:**
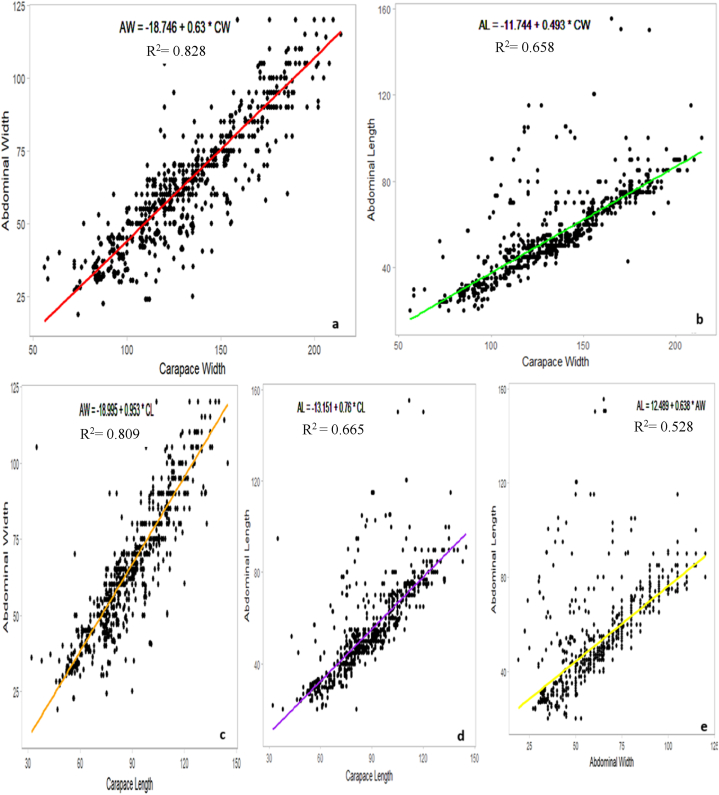
Carapace width-Abdominal width relation (a), Carapace width-Abdominal length relation (b), Carapace length-Abdominal width relation (c), Carapace length-Abdominal length relation (d), Abdominal width -Abdominal length relation (e) of female *S. serrata*.

The interrelationships observed between the different morphological characteristics of females (Fig. 8) provide insights into how the dimensions of certain body parts scale within *S. serrata*. CW had strong correlations with AL and AW, which explained 65.80 % and 82.86 % variations in measurements, respectively. In the same way, CL served as a good predictor for the abdominal dimensions of the crab, explaining 66.59 % and 80.9 % of the variation in AL and AW, respectively. AL and AW were moderately related wherein 52.82 % of the variation in AL was explained by AW. The large R^2^ values and low p-values indicate that all these relationships are significant statistically and explain nearly all the dependent variable variations.

### 3.4 Condition factors

Three types of condition factors, namely Fulton's condition factor (K), the allometric condition factor (K*a*), and the relative weight condition factor (K*n*) were estimated in this study, as listed in Table 7. The mean K values for male crabs were 0.017 ± 0.004 and 0.014 ± 0.003, respectively. K*a* for male crabs ranged from 0.0007 to 0.016; for females, it was slightly higher, ranging between 0.0031 and 0.090. As shown in Table 7, K*n* had the lowest values for female crabs, which varied between 0.113 and 3.205, and for males, it ranged between 0.152 and 3.516.

**Table 7:** Different condition factors and Mean and Standard Deviation of condition factors.

sex	Fulton's condition factor K		Allometric condition factor (K*a*)		Relative condition factor K*n*	
	Range K	Mean K	Range K*a*	Mean K*a*	Range K*n*	Mean K*n*
Female	0.001–0.052	0.014 ± 0.003	0.0031–0.090	0.0286 ± 0.005	0.113–3.205	1.019 ± 0.202
Male	0.002–0.054	0.017 ± 0.004	0.0007–0.016	0.0045 ± 0.0009	0.152–3.516	1.019 ± 0.219
Pooled	0.001–0.054	0.015 ± 0.003	0.001–0.037	0.0105 ± 0.002	0.102–3.608	1.025 ± 0.231

## 4 Discussion

### 4.1 Population structure and size frequency distribution

Size frequency distribution is a statistical method that depicts the size distribution of a population of organisms. The size-frequency distribution of male and female *S. serrata* in the Cochin Estuary demonstrates clear sexual dimorphism in terms of size, with males dominating the larger size classes. Differences in size and weight among populations are common in crabs and are greatly influenced by the sex ratio, food availability, population health, ﬁshing pressure, and genetic variation [17]. The dominant size class represents individuals that are more commonly harvested. In the present study, the dominant size class for male crabs was 126–136 mm and female crabs had modal length classes of116–126 mm. This indicates that the fishery depends on sub-adults of males and females. The CW in the present study ranged from 56 mm to 216 mm, and the largest crab with a 214 mm CW was female. The largest *S. serrata* with a CW of 213 mm was reported in the Korapuzha Estuary, Kerala, and with a size class of 46–215 mm [28]. In *S. ocanica* (the species has been redescribed as *S. serrata* by Ref. [1]), a size range of 61–210 mm was observed in Ashtamudi Lake, Cochin Estuary and Korapuzha Estuary [34], in Andhra Pradesh, India, a size range of 31–134 mm of *S. serrata* was reported [30], with a size range of 15–129 mm in *S. serrata* from the backwaters of Kakinada [27]. The CW of *S. serrata* in Chilika Lagoon is 20–181 mm [35]. The average size and weight of the *S. serrata* population from the Cochin estuary were much larger than those in similar studies conducted in India [27,28,30]. However, the size class of *S. serrata* obtained in the present study was much larger than that in studies from other countries. In Indonesia, adult individuals have carapace widths of 120–200 mm [23]. The CW of *S. serrata* is between 100 and 200 mm in Japan [24] and 78–235 mm in Kotania Bay of the Western Seram District, Maluku Province of Eastern Indonesia [36], and in the Persian Gulf, S. *serrata* varies between 62 and 212 mm [37]. The maximum reported sizes of CW seem to be slightly different in the different regions, with CW 228 mm in Pohnpei [38], 206 mm [39], 209 mm in Australia [40], 200 mm in South Africa [41], 230 mm in Kosrae [42] 120 mm, in male crabs, 132 mm in female crabs Karang Gading Sumatera Utara [43], 130 mm in Inhaca Islands Mozambique [44], and 213 mm in coastal waters of western Seram, Indonesia [45]. This is mainly due to food availability, population health, ﬁshing pressure, and genetic variation [46].

### 4.2 The carapace width-weight relationships (CW-WR)

CW–WR have been employed in the mud crab population as a useful tool to predict W at a certain CW or vice versa. From the CW-WR, the *b* value*,* based on the *b* value growth pattern, can be defined as either isometric growth (*b* = 3), positive allometric (*b* > 3), or negative allometric (*b* < 3), and can also provide insights into how crabs allocate energy to growth during different stages of their life cycle, especially regarding moulting. A common growth pattern can be observed among the crab species, with a tendency for males to be heavier than females [47]. In males and females of *S. serrata*, the '*b*' values during the present study were 3.268 and 2.86, respectively. This shows that male crabs gain weight faster than their CW and that males exhibit positive allometric growth. In females, weight increases slower than males, exhibiting slightly negative growth allometry. In all earlier reports of CW-WR, *Scylla* sp. displayed positive growth allometry(*b* > 3) for males, while females displayed negative growth allometry (*b* < 3) [26]. The *b* value of *S. oceanica* (currently *S. serrata*) from Ashtamudi Lake, Cochin backwater, and Korapuzha Estuary for males, 3.1185, 3.142 and 3.0771 respectively, whereas in females, 2.939, 2.999 and 2.935 respectively [34]. From the Chilka Lagoon, Odisha, India, *b* was higher in males [29]. Studies conducted in Andhra Pradesh, India, also showed that the *b-value* for the relationship in males was 3.0426 (positive allometry). In contrast, in females, the b value was 2.775 (negative allometry); for pooled samples, the *b* value was 2.9210 [30]. In North Kalimantan, Indonesia, males showed positive allometric development, and females showed allometric growth with negative expression [25]. However, in the Hooghly-Matlah Estuary in West Bengal, the CW-WR is W = 0.0006 CW^2.665^ for males and W = 0.001 CW^2.561^ for females, with males showing a slightly higher trend and negative allometry [48]. The CW–W relationship of crab populations varies temporally and spatially [18]. As in many other brachyuran crab species, adult male mud crabs might attain twice the weight of adult females of the same size because of their larger chelae [49,50]. This ensures the ability to successfully defend females during and after copulation [44]. In juvenile male crabs, *b* = 2.489, indicating negative allometric growth. However, in sub-adults and adults, *b* = 3.036 and *b* = 3.407, respectively, indicated positive allometric growth. In juvenile female crabs, *b* = 1.61, it shows negative allometric growth; in sub-adults and adults, *b* = 2.861 and *b* = 2.774, respectively, indicating negative allometric growth. Crabs grow and mature through moulting [51]. Moulting introduces variability in the *b-value* of the allometric growth equation. The juvenile stage is a critical phase, in which the crab undergoes significant growth before reaching maturity [52]. The growth rate at this stage is often faster than that of adult crabs [51]. The *b* value of juvenile male crabs is b = 2.489, which shows that the crab increased its CW faster than its weight; juvenile mud crabs moult frequently, which results in a high growth rate, but there is a decrease in moult frequency with an increase in age [51]. In an experimental study [53], the mean growths in weight of the *S. serrata* were 21.66 ± 1.94g for females, 28.00 ± 7.02g for males and 19.91 ± 2.25g for immature, showing that the weight gain in the juvenile stage is slow. The differences in sex-specific growth traits increased in larger crabs (CW = 70–80 mm) [47]. In the present study, the smallest female crab had a CW of 54 mm and the smallest male crab had a CW of 73 mm; therefore, there was a large difference in the *b* values of juvenile males and females. In the sub-adult and adult stages, the W of the male crab was more likely to be influenced by cheliped weight by approximately 45 %, whereas the female cheliped only contributed 22 % of its body weight [54]. Chelae size increased with the growth of Scylla sp [39]. For example, in the 100–150 mm CW, the male chelae weighed 16.6 g, but the female weighed 13.8 g. As the crab matured (150–200 mm CW), the male chelae crusher weighed 28.1 g, while the females weighed 11.3 g [47]. The development of male claws, which are approximately 2.5 times higher than the female claw [47], and because of this the difference in the *b* value is observed in males and females. Depending on the biological, temporal, and sampling factors [55] food availability [21] and water quality [56,57] the growth of species may differ for different populations of the same species or the same population over various years.

### 4.3 Interrelationships between different morphometric characters

The interrelationships between various morphometric characteristics of male and female *S. serrata* provide valuable insights into their growth patterns and body scaling. For males, CW exhibited strong correlations with ChL and ChW. Likewise, CL exhibited strong predictive power for ChL and ChW. In females, *S. serrata*. CW had strong correlations with AL and AW, explaining 65.80 % and 82.86 % of the variations in these measurements, respectively. Similarly, CL served as a good predictor for the abdominal dimensions of the crab, explaining 66.59 % and 80.9 % of the variation in AL and AW, respectively, indicating that carapace dimensions are reliable indicators of abdominal dimensions. CW and CL are strong indicators of chelar propodus dimensions and abdominal dimensions, indicating that as crabs increase in size, their claws and other morphometric features scale proportionally with carapace measurements. This suggests a consistent and predictable relationship between body size and limb growth, reflecting a coordinated developmental strategy. Most of the studies focus on the CW-W relationship and interrelationships between different morphometric characters are not studied.

### 4.4 Condition factors

#### 4.4.1 Fulton's condition factor (K)

The mean K values for the male crabs were 0.017 ± 0.004 and 0.014 ± 0.003 for the female crabs. These values indicate that males generally have a slightly higher condition factor than females, suggesting that males are in marginally better condition than females. The condition factor is an important metric in assessing the overall well-being of crabs, reflecting aspects like nutritional status and reproductive health. The mean K value of *S. serrata* from Chilka Lagoon India varied from 0.0350 to 0.0719 in males and from 0.0368 to 0.0671 in females [29]. The lower K values observed in the present study may be attributed to environmental conditions, food availability, or other ecological factor.

#### 4.4.2 Allometric condition factor (K*a*)

The allometric condition factor (K*a*) is typically employed when a species shows allometric growth or when the *b*-value is computed using sufﬁcient data to reduce the calculation error [22](Bagenal& Tesch, 1978).To eliminate the allometric effect from the estimation of crab condition, the condition factor K*a* is used [58]. It is used to determine the feeding activity of ﬁsh [59] and as an indicator of feeding intensity in laboratory experiments [60]. The K*a* value reflects the relationship between the W and CW. The present study K*a* for male crabs ranged from 0.0007 to 0.016; for females, it was higher and ranged between 0.0031 and 0.090. *S. serrata* has *b* values for males were 3.268 and for females 2.86, i.e. *b*≠3. Therefore, the species has an allometric growth pattern, and K*a* is probably more appropriate, where the differences in the condition factor are directly related to differences in weight and food intensity [18]. The growth patterns and weight differences between male and female crabs influence their K*a* values. Relative to their CW, female crabs have a higher weight-to-width ratio. Female crabs often develop their ovaries they become much heavier, which adds to their weight without significantly increasing their carapace width. This contributes to a higher K*a* value. A higher K*a* value generally indicates better condition or robustness. The K*a* value is higher than the K value in the present study, indicating that the crabs are in better condition when accounting for their allometric growth pattern. K*a* of the mud crab is not much studied.

#### 4.4.3 Relative weight condition factor (K*n*)

The relative weight condition factor (K*n*), varied between 0.113 and 3.205; for males, it ranged between 0.152 and 3.516 for females and the average K*n* values for males and females were 1.019 and for pooled 1.025. Compared to the present study the K*n* values for both male and female mud crabs were lower in Coringa Wildlife Sanctuary in Andhra Pradesh, India ranging from 0.65 to 1.37 [30]. Also, From the Chilka Lagoon, India the K*n* values of *S. serrata* ranged from 0.83 to 1.21in males and 0.84–1.13 in females [29]. K*n* values of the present study were lowest for female crabs, this is similar to the findings of [29,30]. From the coastal waters of Bangladesh, the Kn values (they represent it as K_R_) of *S. serrata* ranged from 0.728 to 1.122 [31]. From the Karang Gading Sumatera Utara, in *S. serrata* male and female the condition factor ranges from 1.37 to 2.56, 1.05 to 2.68 respectively. With an average of 1.54. The K*n* value around 1 indicates that food availability and other physicochemical characteristics are likely to be optimum [19]. The K*n* > 1 indicates that the crabs are in good condition, abundant food and favourable environmental conditions. Male crabs typically grow larger and faster, which can result in higher K*n* values, For growth the moulting process is crucial, During the breeding season, females moult less frequently allocate resources to egg production and spawning, and also migrate to the oceanic condition for spawning, which can reduce their overall body condition and lead to lower K*n* compared to males, in contrast, males remain in more stable environments, which can support better overall conditions and higher K*n* values [51]. Differences in feeding behaviours between males and females can also contribute to variations in K*n* values. Males may have more consistent access to food resources, while females might experience periods of reduced feeding during spawning migrations [51]. In most of the CW-WRs studies only K is calculated, it is preferable to study K, K*a*, and K*n* together. That is not a rule, only if K > 1, the better will be the condition of the crab.

## 5 Conclusion

This study provides comprehensive insights into the population structure, growth patterns, and condition factors of *S. serrata* in the Cochin estuary. The size-frequency distributions revealed key information about the population structure, indicating the prevalence of juveniles, sub-adults, and adults within the estuary. The CW-WR demonstrated distinct growth patterns across different life stages and sexes. The CW-WR in male crabs demonstrated a predominantly positive allometric growth pattern and a negative allometric pattern in females. In male crabs, the CW-WR indicated negative allometric growth in juveniles and positive allometric growth in sub-adults and adults. The CW-WR showed negative allometric growth across all stages in female crabs. These findings highlight variations in growth dynamics between sexes and life stages. The condition factors (K, K*a,* K*n*) highlighted the overall health and well-being of the crab population, providing critical data for sustainable fishery management. These findings contribute new data to the limited knowledge of *S. serrata* in Indian waters and underscore the importance of understanding local environmental and biological dynamics. The results are expected to aid researchers, conservation biologists, and policymakers in stock assessments and the development of evidence-based strategies for the conservation and sustainable management of mud crab fisheries in the Cochin estuary. The results also include some variations of the previous study related to the change in biological, physiological, and environmental factors. It is hoped that the results of this research will have a useful effect on the stock assessment of mud crabs.

## CRediT authorship contribution statement

**K.A. Aneesa:** Writing – original draft, Software, Methodology, Investigation, Formal analysis, Conceptualization. **E.R. Chaithanya:** Writing – review & editing, Validation, Supervision, Conceptualization. **Sijo P. Varghese:** Writing – review & editing, Validation, Supervision, Conceptualization.

## Ethical statement

No licensing was required to collect the specimens because it is a common edible species, and the sampling location is the common landing area. All the measurements were taken From the Live crabs, no crabs were killed for this particular study.

## Data availability statement

Raw data were generated from the sampling. Derived data from sampling that support the findings of this study are available from the corresponding author SPV on request.

## Declaration of competing interest

The authors declare that they have no known competing financial interests or personal relationships that could have appeared to influence the work reported in this paper.
